# Massive subcutaneous filariosis by *Filaria martis* in beech marten (*Martes foina*) in Italy

**DOI:** 10.1016/j.parepi.2024.e00406

**Published:** 2024-12-31

**Authors:** Giovanni Sgroi, Ranju Ravindran Santhakumar Manoj, Ettore Napoli, Nicola D'Alessio, Maria Gabriella Lucibelli, Claudio de Martinis, Esterina De Carlo, Peyman Khademi, Alireza Sazmand, Vincenzo Veneziano

**Affiliations:** a Animal Health Department, Experimental Zooprophylactic Institute of southern Italy, 2 Via della Salute, 80055 Portici, Naples, Italy; b Population Medicine and Diagnostics Department, Cornell University, 616 Thurston Avenue, 14853 Ithaca, NY, USA; c Veterinary Department, Messina University, 98168 Messina, Italy; d Microbiology and Food Hygiene Department, Lorestan University, 44316-68151 Khorramabad, Iran; e Pathobiology Department, Bu-Ali Sina University, 6517658978 Hamedan, Iran; f Veterinary Medicine and Animal Production Department, Naples University, 8 Via Mezzocannone, 80138 Naples, Italy

**Keywords:** *Filaria*, Filarioid, *Ixodes*, Nematode, Vector-borne, Wildlife

## Abstract

The beech marten (*Martes foina*) is a small-size mustelid endangered according to the IUCN Red List. Despite the plethora of parasites potentially affecting its population decline, subcutaneous filarioids are occasionally reported in martens and their competent arthropod vectors are to date unknown. Therefore, from January 2023 to August 2024, this study investigated the presence of subcutaneous filarioids and ectoparasites of road-killed beech martens (*n* = 7) from southwestern Italy. One marten (14.3 %, 95 % CI: 2.6–51.3) was massively infected with subcutaneous filarioids, i.e., 18 specimens (11 males and 7 females) in the dorso-posterior region, where mild redness and sloughing of skin were found. All the filarioids were identified as *Filaria martis* via morphology and Sanger sequencing of the *cytochrome c oxidase* subunit I (*cox*1) gene that revealed a single sequence type (GenBank accession number PQ034642) having 100 % nucleotide identity with those available in the literature. The phylogenetic analysis displayed a bootstrap value of 100 % between the *cox*1 sequences of *F. martis* of this study and those of beech martens from Italy and European minks from Spain. Haemolymph, gut, and salivary glands of the *Ixodes ricinus* ticks collected from the infected marten scored negative for *F. martis* larvae and DNA by dissection and PCR, respectively. This study reported for the first time *F. martis* subcutaneous filariosis in southwestern Italy, previously outlined only in northern and southeastern areas, indicating the circulation of this poorly investigated filarioid. Knowledge of the competent vectors involved in the biological cycle of *F. martis* requires further experimental studies.

## 1 Introduction

The beech marten or stone marten, *Martes foina*, is a native mustelid found across much of Europe and some parts of Asia, closely related to the slightly larger pine marten, *Martes martes*, and classified as “least concern” on the red list of threatened species by the International Union for Conservation of Nature (IUCN) (Abramov et al., 2016). Beech martens show flexible habitat preferences, being able to live in environments ranging from deciduous forests and forest edges to open rocky slopes, as well as in suburban and urban areas (Rondinini and Boitani, 2002; Santos and Santos-Reis, 2010; Zub et al., 2018; Fonda et al., 2021). Primarily nocturnal, these agile hunters have a voracious appetite, feeding on bird eggs, insects, worms, berries, small rodents, and amphibians (Abramov et al., 2016). This diverse diet and habitat may increase the likelihood of beech martens as definitive or intermediate hosts for various agents, enhancing the risk of parasite transmission among wildlife, domestic animals, and humans. Indeed, beech martens have been ascertained to host a plethora of parasitic agents in Europe, such as urinary bladder capillariids (e.g., *Capillaria plica* - syn. *Pearsonema plica*), eyeworm spirurids (e.g., *Thelazia callipaeda*), nasal and tracheobronchial metastongylids (e.g., *Skrjabingylus petrowi*, *Crenosoma vulpis*), muscular adenophoreans and protozoa (e.g., *Trichinella spiralis*, *Sarcocysts lutrae*), intestinal protozoa (e.g., *Cryptosporidium ditrichi*) and subcutaneous filarioids (e.g., *Dirofilaria repens*, *Filaria martis*) (Otranto et al., 2007; Miterpáková et al., 2013; Heddergott et al., 2015; Prakas et al., 2018; Petersenf et al., 2018; do Vale et al., 2019; Deak et al., 2023; Perec-Matysiak et al., 2023). Among these, *F. martis* (class Chromadorea, order Rhabditida) is the least studied subcutaneous nematode species although able to infect different hosts of the families Mephitidae (e.g., striped skunks, *Mephitis mephitis*) (Worley, 1961), Pedetidae (e.g., south African springhares, *Pedetes caffer*) (Chabaud and Mohammad, 1989; Anderson, 2000), and Mustelidae. In mustelids, limited reports are available worldwide on this poorly known subcutaneous filariosis-causing parasite, to date outlined only in beech martens from northern (Anderson, 1960; Casiraghi et al., 2001) and southeastern parts of Italy (Otranto et al., 2007), European minks (*Mustela lutreola*) from northern Spain (Torres et al., 2016), and American badgers (*Taxidea taxus*) from the United States of America (Worley, 1961).

Herein, we report a case of massive infection by *F. martis* in a beech marten from southwestern Italy, using morphological and molecular analyses, including phylogenetic insights.

## 2 Materials and methods

### 2.1 Study area and sampling

From January 2023 to August 2024, within a health monitoring plan of wildlife (authorization no. DD 210-B7 DPAR), carcasses of beech martens (*n* = 7) were retrieved deceased after road accidents in different rural and peri-urban areas of southwestern Italy (Campania region). The time-lapse from death to the examination of martens could not be estimated; however, only fresh carcasses were examined. An information form was compiled in the field with animal data such as sex and collection province (Table 1). Field activities were carried out in collaboration with “trained persons” (i.e., hunters, forest rangers, and wildlife conservationists) who previously attended a theoretical and practical course on the biology, ecology, and health and hygiene of wildlife according to the Reg. EU 853/2004 (Sgroi et al., 2023). Once found, only fresh carcasses were considered for sampling and immediately collected in specific bags inside refrigerated boxes to allow the collection of any live ectoparasites present. The carcasses were then delivered to the Wildlife Disease Unit, Animal Health Department, of the Experimental Zooprophylactic Institute of southern Italy (Naples, Italy) for a full necropsy.

**Table 1:** Number and percentage of beech martens (*n* = 7) investigated for subcutaneous filarioids in southwestern Italy in 2023–2024, according to sex, age class, year, and collection province.

	Number (percentage)
**Sex**	
Male	4 (57.1 %)
Female	3 (42.9 %)
**Age**	
Young	0 (−)
Juvenile	3 (42.9 %)
Adult	4 (57.1 %)
**Year**	
2023	5 (71.4 %)
2024	2 (28.6 %)
**Province**	
Avellino	2 (28.6 %)
Benevento	3 (42.9 %)
Salerno	2 (28.6 %)

### 2.2 Necropsy, filarioid and tick collection

Upon delivery to the lab, the animal age class was determined in three different groups (i.e., young, juvenile, and adult), according to the skull feature evaluation (Albayrak et al., 2008). All the collected carcasses were carefully examined for the presence of ectoparasites, and when found were classified according to sex (female or male), developmental stage (larva, nymph, adult), and feeding status (fed or unfed), then identified morphologically using valid keys (Estrada-Peña et al., 2017). The ticks were kept alive for one day at 25 °C and 75 % relative humidity and then dissected to set up separate smears of haemolymph, gut, and salivary glands on glass slides. The slides were examined under an optical microscope (Leica DMLB2, Leica Microsystems, Wetzlar, Germany) equipped with the Leica LAS version 4.5.0 software (Leica Application Suit, Leica Microsystems, Wetzlar, Germany) to assess the presence of any filarioid stage. Then a complete skinning was run to assess the presence of subcutaneous filarioids. Helminth specimens were collected in 96 % ethanol and delivered to the Parasitology Units of the Universities of Messina and Naples for morphometric and molecular analyses.

### 2.3 Morphometric analysis of filarioids

Each nematode isolated after skinning was transferred into a Petri dish, rinsed twice with a saline solution to remove any debris, and subsequently fixed in 70 % ethanol. In order to display their structural features, the specimens were treated with glycerine for 2 h to clarify the tissues, and then mounted on temporary slides in glycerine. Structural features of the filarioids were evaluated via optical light microscopy and the software mentioned above with magnification ranging from 5× to 100×; all measurements obtained were compared with those available in literature (Otranto et al., 2007; Chabaud and Mohammad, 1989; Anderson, 1960) to identify the nematodes at species level.

### 2.4 DNA extraction, PCR, and sequencing

DNA was extracted from the filarioids and ticks by using twenty-five milligrams of tissue individually homogenized by TissueLyser III (Qiagen, Hilden, Germany) in sterile phosphate-buffered saline (PBS) with two 4.8 mm glass beads (Diatech Lab Line, Salerno, Italy). Each DNA extraction session included a negative extraction control (i.e., equal volume of RNase/DNase free water instead of DNA extraction elute). From 200 μL of homogenized sample, extraction of nucleic acid was obtained using the commercial kit QIAampDNA Blood & Tissue (Qiagen, Hilden, Germany), following the manufacturer's instructions.

An endpoint conventional PCR protocol was run to amplify a 689 bp fragment of filarioid helminths cytochrome *c* oxidase subunit I gene (*cox*1) with primers NTF: 5′-TGATTGGTGGTTTTGGTAA-3′ and NTR: 5’-GATATTGATACTCGTACTTAT-3′ (Casiraghi et al., 2001). Reactions were run with HotStart Master Mix (Qiagen, Hilden, Germany), 1 μM of each primer, and ≈100 ng of DNA template measured with the Biofhotometer plus (Eppendorf, Hamburg, Germany) in a final volume of 25 μL, including 5 μL DNA template. Thermocycling conditions by Otranto et al. (Otranto et al., 2007) were modified as follows: initial denaturation at 95 °C for 15 min, followed by 40 cycles of denaturation at 95 °C for 60 s, annealing at 48 °C for 60 s, extension at 72 °C for 60 s and final extension at 72 °C for 7 min. Amplicons obtained were purified and Sanger sequenced in both directions using the same primers as for PCR by a commercial company (Eurofins Genomics, Ebersberg, Germany). Sequences were aligned, trimmed, and edited by using BioEdit software (Hall, 1999) and compared with those available in the GenBank database by the Basic Local Alignment Search Tool (BLAST; https://blast.ncbi.nlm.nih.gov/Blast.cgi).

### 2.5 Phylogenetic analysis

The phylogenetic analysis was based on 602 bp *cox*1 gene sequences of *F. martis* in beech marten of this study with sequences of cutaneous filarioid species from different hosts and countries. The phylogenetic analysis was inferred by using the maximum-likelihood (ML) method based on the General Time Reversible model and gamma distribution used to assess evolutionary rate differences among sites (+G) selected by best-fit model (Nei and Kumar, 2000). Evolutionary analyses were conducted on 8000 bootstrap replications using the MEGA X software (Kumar et al., 2018). The homologous sequence of *Dracunculus medinensis* was used as an out-group (accession number: HQ216219).

### 2.6 Statistical analysis

An exact binomial 95 % confidence interval (95 % CI) was established for the proportion of infection herein found, by using the online software Epitools-Epidemiological Calculators (Sergeant, 2018). The distribution of GPS collection sites of positive and negative martens in the study area was obtained with QGIS software (version 3.10.2 with GRASS 7.8.2).

## 3 Results

**Fig. 1:**
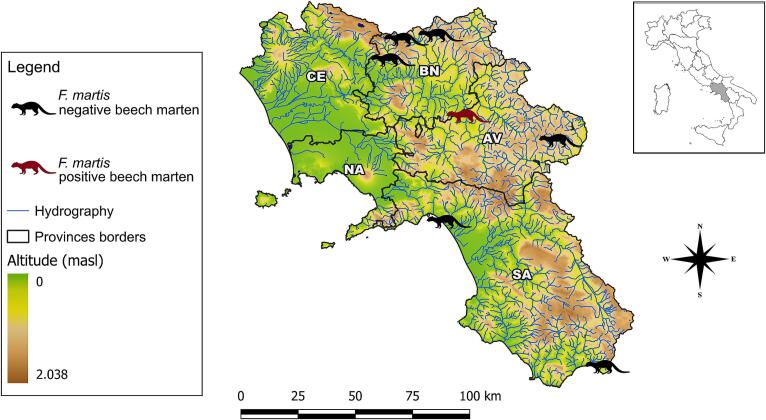
Distribution of *Filaria martis-*positive and negative beech martens in the study area, according to province borders of the study area (i.e., Avellino, AV; Benevento, BN; Benevento; Caserta, CE; Napoli, NA, Salerno, SA).

On necropsy, one beech marten out of 7 (i.e., 14.3 %, 95 % CI: 2.6–51.3) tested positive for *F. martis* by the combined morphological/molecular approach. The distribution of positive and negative animals in the study area is shown in Fig. 1.

**Fig. 2:**
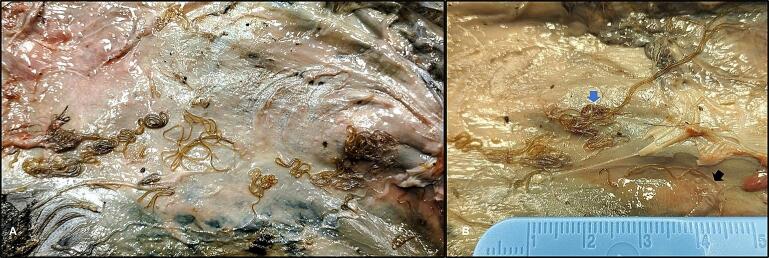
Macroscopic appearance of *Filaria martis* in subcutaneous tissue of beech marten. (A) Subcutaneous redness associated with a massive *F. martis* infection. (B) Detail of *F. martis* free (blue arrow) and incapsulated (black arrow) in the subcutaneous tissue.

During the necropsy, 18 helminths were found in the subcutaneous tissue in the dorso-posterior body region of one marten. No gross pathological signs related to the infection were observed except a mild skin sloughing and subcutaneous redness (Fig. 2A). All helminths collected were morphometrically identified as 11 (61.1 %) males and 6 (38.9 %) females of adult *F. martis* specimens. Most of the recovered nematodes were free in the subcutaneous tissue (*n* = 15) whereas few specimens incarcerated in subcutaneous membranous capsules (*n* = 2) (Fig. 2B).

**Fig. 3:**
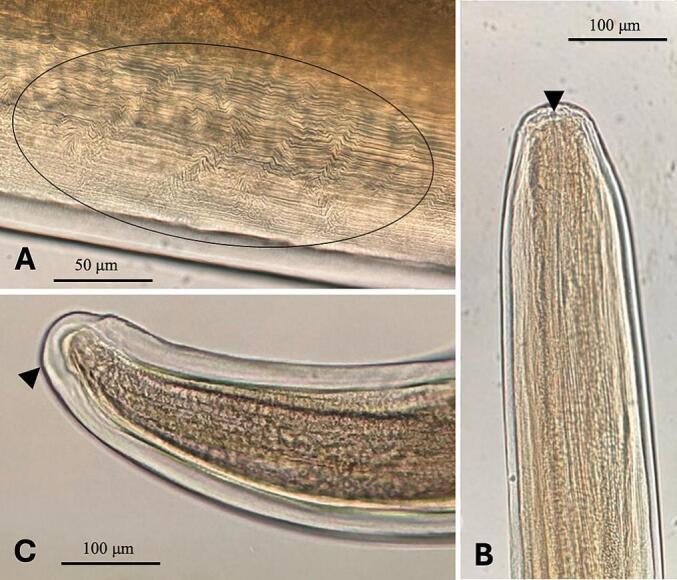
Microscopic appearance of *F. martis* specimens. (A) Striated cuticle (circle). (B) Cephalic end with a chitinous pre-esophageal ring (black arrowhead). (C) Posterior end with smooth caudal tips without spines (black arrowhead).

The parasites were yellowish, long and thin, attenuated at the cephalic and caudal ends. The cuticle was striated (Fig. 3A) and the cephalic end characterized by 4 pairs of conoidal papillae and a chitinous pre-esophageal ring (Fig. 3B). Females measured 160–193 (173 ± 7.50) mm in length and 0.29–0.38 (0.33 ± 0.03) mm in width, while males 80–93 (85.11 ± 6.37) mm in length and 0.23–0.33 (0.29 ± 0.01) mm in width. Muscular and glandular portions of the esophagus were clearly separated in females, and slightly divided in males. In females, the vulva was located at 35.24 ± 0.69 μm next to the mouth opening, and the posterior end had smooth caudal tips without spines (Fig. 3C). Males had curved tails with caudal *alae* and asymmetrical *spicula* of 0.48 ± 0.10 mm on the left versus 0.18 ± 0.03 mm on the right.

**Fig. 4:**
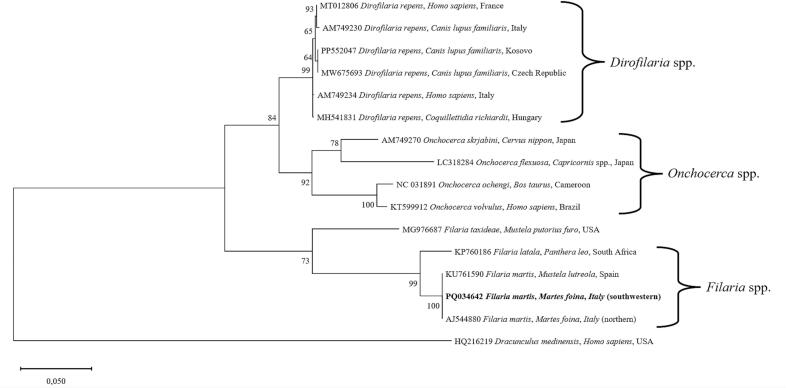
Phylogenetic network on 602 bp *cox*1 gene sequences of *F. martis* found in this study with cutaneous filarioid species from different hosts and countries. The phylogeny was inferred with the maximum-likelihood method based on General Time Reversible and gamma distribution was used to model evolutionary rate differences among sites (+G) selected by best-fit model (Nei and Kumar, 2000). Evolutionary analyses were conducted on 8000 bootstrap replications using the MEGA X software (Kumar et al., 2018). Homologous sequence of *Dracunculus medinensis* was used as out-group.

The morphological identification of *F. martis* was confirmed by sequencing that revealed all the specimens herein tested as a single sequence type having 100 % query coverage and 100 % nucleotide identity with *F. martis* sequences available in GenBank. The phylogenetic analysis displayed 100 % bootstrap value among the *cox*1 gene partial sequences of *F. martis* from this study and those of beech marten from Italy (AJ544880) and European mink from Spain (KU761590) (Fig. 4). The sequence type obtained in this study was submitted to GenBank under accession number PQ034642.

The marten positive for *F. martis* was the only animal infected by ectoparasites i.e. two engorged female *Ixodes ricinus* ticks. Smears of haemolymph, gut, and salivary glands prepared from the ticks scored negative for any filarioid.

## 4 Discussion

This study reports the presence of *F. martis* in southwestern Italy, to date reported only in beech martens from northern (Anderson, 1960; Casiraghi et al., 2001) and southeastern parts of the country (Otranto et al., 2007).

Although lower than that in southeastern Italy (i.e., 37.9 % out of *n* = 29) (Otranto et al., 2007), the prevalence of *F. martis* herein found is not negligible (14.3 %). However, given the small sample size, large-scale surveys are needed on this mustelid for obtaining epidemiological insights. In fact, the wide CI obtained (i.e., 2.6–51.3 %) is a consequence of the small sample size indicating as meaningful conclusions on the real prevalence and geographic distribution of *F. martis* should be considered with caution.

The low number of reports on *F. martis* in the literature could be due to the scant availability of these mustelids for necroscopic examination (Lovari and Rolando, 2004) as well as the uncommon practice of skinning animal carcasses that, taken together, make the collection of epidemiological data on the distribution of this filarioid difficult to obtain.

The massive infection in this study (18 specimens in one individual) is in accordance with the mean parasitic burden previously found by Otranto et al. (Otranto et al., 2007) who reported a value of 21 *F. martis* specimens per positive beech marten (*minimum* 1 - *maximum* 48). In addition, filarioids found in this study were mostly free in the subcutaneous tissue, in accordance with previous studies showing a low number of specimens localized in membranous capsules (Otranto et al., 2007; Torres et al., 2016) which have been suggested as a site where gravid females could stay in a transient stage of their life cycle before releasing parasitic eggs (Worley, 1961).

The finding of mild sloughing and redness at the subcutaneous tissue in conjunction with filarioids is in accordance with previous studies (Otranto et al., 2007; Torres et al., 2016) and suggests the occurrence of a dermatitis associated with the infection, although a clear evaluation of signs ascribable to the parasites is not possible in the present study considering the origin of the animal from road accidents. Therefore, further studies based on histopathological tools on the pathogenicity of the parasite on beech marten are desiderable.The morphometric analysis of the *F. martis* specimens of this study are consistent with those previously observed (Otranto et al., 2007; Chabaud and Mohammad, 1989; Anderson, 1960; Torres et al., 2016), suggesting a low morphological variability of this filarioid species.

Both, the high nucleotide identity (i.e., 100 %) and bootstrap value (i.e., 100 %) among the *cox*1 sequence in this study and those of the literature would lead to ruling out the circulation of different parasite strains, although more research is needed to confirm or discard this hypothesis. In addition, the well-defined phylogenetic clustering of several taxa of the Filarioidea superfamily herein obtained underscores the high ability of *cox*1 sequencing to differentiate among subcutaneous filarioids (Lefoulon et al., 2015; Laidoudi et al., 2020; Dos Santos et al., 2022).

The absence of *F. martis* larvae and DNA in the two *I. ricinus* collected from the positive animal in this study does not exclude the role of this tick species as a competent vector of the filarioid. Indeed, the transmission of *F. martis* by ticks, fleas, or mosquitoes is likely considering that ticks (i.e., *Rhipicephalus sanguineus* sensu lato), fleas (i.e., *Ctenocephalides canis*, *Ctenocephalides felis*), and mosquitoes (i.e., *Aedes* spp., *Anopheles* spp., *Culex* spp.) are proven vectors for other subcutaneous filarioids such as *Cercopithifilaria bainae*, *Acanthocheilonema reconditum,* and *Dirofilaria repens*, respectively (Taylor et al., 2022). These ectoparasites are widespread in the Mediterranean basin (Bezerra-Santos et al., 2021; Pacifico et al., 2021; Sgroi et al., 2021; Sgroi et al., 2022). Unfortunately, experimental studies on arthropods collected from animal carcasses are often unavailable because following the reduction in carcass temperature after death these sucking ectoparasites abandon the host animals (Torres et al., 2016). Alternately, the transmission of *F. martis* could be similar to some other subcutaneous filarioids, such as *Parafilaria multipapillosa* (Otranto et al., 2007) and *Filaria taxidae* (Torres et al., 2016; Mulreany et al., 2018), in which case, deposited helminth larvae (microfilariae) on skin wounds of infected animals are ingested by Muscidae flies *Haematobia* spp., develop to the infective L_3_ stage in the fly, and again deposed on skin wounds of healthy animals (Marchiondo et al., 2020).

## 5 Conclusion

The data above showed the presence of a single sequence type of *F. martis* in martens of southwestern Italy. Further research is needed to clarify the epidemiology of *F. martis*, revealing the competent vectors involved in the biological cycle and potential additional hosts of this filarial nematode.

## Funding

This study was supported by 10.13039/501100003852Regione Campania (Grant No. DD 257-2022).

## Ethical standards

The animal carcasses in this study were analysed under the frame of a health monitoring plan of wildlife authorized by Regione Campania (Authorization No. DD 257-2022).

## CRediT authorship contribution statement

**Giovanni Sgroi:** Writing – original draft, Conceptualization. **Ranju Ravindran Santhakumar Manoj:** Writing – original draft. **Ettore Napoli:** Methodology, Investigation. **Nicola D'Alessio:** Resources, Formal analysis. **Maria Gabriella Lucibelli:** Software, Methodology. **Claudio de Martinis:** Data curation. **Esterina De Carlo:** Validation. **Peyman Khademi:** Writing – review & editing. **Alireza Sazmand:** Writing – review & editing. **Vincenzo Veneziano:** Supervision, Project administration.

## Declaration of competing interest

The authors declare no competing interests.

## Data Availability

The datasets analysed during the current study are available from the corresponding author.
